# Best Practices for Living Labs When Studying Older Adults Living in Rural Communities

**DOI:** 10.1093/geroni/igab046.3673

**Published:** 2021-12-17

**Authors:** Ashley Nakagawa, Shannon Freeman, Alanna Koopmans, Chris Ross, Richard McAloney

**Affiliations:** 1 University of Northern British Columbia, Honolulu, Hawaii, United States; 2 University of Northern British Columbia, Prince George, British Columbia, Canada; 3 UNBC, University of Northern British Columbia, British Columbia, Canada; 4 University of Northern British Columbia, University of Northern British Columbia, British Columbia, Canada

## Abstract

There are two core concepts that make living labs distinguishable: involvement of users as co-creators and evaluation in a real-world setting. Living labs increase the potential for product acceptance and adoption due to testing and tailoring with target users. Currently, there is a lack of a universally accepted guideline for best practices. The objective of this review is to identify the best practices of living labs that can be recognized by the scientific community and followed in future labs. A 5-stage scoping review, following Arksey and O’Malley’s (2005) framework, was used to map out the coverage of different aspects of living lab methodology. A systematic search for articles involving living lab framework and older adults published between 2016-2021, was conducted in seven databases. Nine articles were included after review, the majority of which were published in health journals and were from Italy and the United States. An overview of consistent user involvement in the innovation process, real-world testing vs. laboratory testing, and participant scope findings will be shared. Multiple rounds of user feedback, real-world testing, and a small but diverse participant group were the most successful in increasing positive sentiments about the products tested in a living lab environment. The lack of published articles on living lab frameworks studying older adults indicate a gap in the literature. Creating a universally accepted definition for living labs and guidelines for best practices will allow for scientific validity and comparisons of studies and may increase the use and popularity of living labs.

